# Early Menarche and Hypertension Among Postmenopausal Women: The Mediating Role of Obesity

**DOI:** 10.3390/epidemiologia6040086

**Published:** 2025-12-02

**Authors:** Eunice Carolina Ibáñez-García, Mónica Alethia Cureño-Díaz, María Alicia Mejía-Blanquel, Ana Cristina Castañeda-Márquez, Ahidée Leyva-López, Yaneth Citlalli Orbe Orihuela, Miguel Trujillo-Martínez, Ricardo Castrejón-Salgado, Erick Ordoñez-Villordo, José Ángel Hernández-Mariano

**Affiliations:** 1 Department of Epidemiological Surveillance, Hospital Juarez of Mexico, Mexico City 07760, Mexico; 2 Department of Institutional Intelligence in Oncological Health, National Institute of Cancerology, Mexico City 14080, Mexico; 3 Department of Research, Hospital Juarez of Mexico, Mexico City 07760, Mexico; 4 Scientific Research Institute, Juarez University of the State of Durango, Durango 34000, Mexico; 5 Center for Population Health Research, National Institute of Public Health, Cuernavaca 62100, Mexico; 6 Center for Research on Infectious Diseases, National Institute of Public Health, Cuernavaca 62100, Mexico; 7 General Hospital with Family Medicine Unit Number 7, Mexican Social Security Institute, Cuautla 62740, Mexico; 8 Health Research Coordination, Mexican Social Security Institute, Cuernavaca 62000, Mexico; 9 Faculty of Medicine, Puebla State University of Health, Puebla 72000, Mexico

**Keywords:** menarche, postmenopause, obesity, hypertension

## Abstract

Background/Objectives: Cardiovascular diseases are the leading cause of mortality worldwide and are strongly influenced by obesity and hypertension. Age at menarche has been proposed as an early marker of cardiometabolic risk, but evidence in postmenopausal women is inconsistent, particularly in Mexico. We aimed to evaluate the association between early menarche and obesity and hypertension in postmenopausal women, and to examine whether obesity mediates this relationship. Methods: We conducted a cross-sectional study based on a retrospective review of 462 medical records of postmenopausal women who attended a tertiary hospital in Mexico City between January 2023 and August 2024. Early menarche was defined as <12 years. Obesity and hypertension were identified from records. Associations were estimated using Poisson regression with robust variance to obtain prevalence ratios (PRs) and 95% confidence intervals (CIs). We assessed effect modification by age at menopause and conducted a mediation analysis under the counterfactual framework to estimate the proportion of the menarche–hypertension association explained by obesity. Results: Early menarche was associated with a higher prevalence of obesity (PR = 1.36, 95% CI = 1.08–1.70) and hypertension (PR = 1.34, 95% CI = 1.06–1.71). Associations were stronger among women with menopause at ≤45 years. Mediation analysis indicated that obesity explained 61.6% of this relationship, with a significant indirect effect (PR = 1.18, 95% CI = 1.05–1.33). Conclusions: Early menarche was independently associated with obesity and hypertension in postmenopausal women, with obesity acting as a potential intermediary factor. Given the cross-sectional design, causality cannot be established, but the associations observed are biologically and temporally coherent.

## 1. Introduction

Cardiovascular diseases are the leading cause of death worldwide, and Mexico is no exception. In 2019 alone, more than 24% of deaths recorded in the country were attributed to these diseases [1]. The burden of these diseases is closely related to preventable cardiometabolic conditions such as obesity and hypertension [2,3]. In particular, hypertension affects 42% of Mexican adult women and constitutes a significant risk factor for coronary artery disease, heart failure, and stroke [4,5].

Obesity is a key determinant in the genesis of hypertension and the progression of cardiovascular diseases [6]. Its prevalence has increased steadily in recent decades [7,8] and disproportionately affects women, especially during hormonal transitions such as menopause. During this period, endocrine changes promote the accumulation of visceral fat and insulin resistance, which, in turn, amplify cardiometabolic risk [9].

An imbalance between caloric intake and energy expenditure is a well-documented mechanism in the development of obesity [10]. However, both obesity and hypertension have a multifactorial etiology. In this regard, reproductive variables have gained increasing attention. Among them, age at menarche has been proposed as an early marker of cardiometabolic risk, as it signals the onset of reproductive maturation and determines the duration of exposure to estrogens and other endocrine changes throughout life [11]. Menarche is considered early when it occurs before age 12 and late when it occurs after age 15 [12,13].

Beyond these biological mechanisms, epidemiological findings have been inconsistent. Some studies report that early menarche is linked to a higher risk of obesity and hypertension, likely due to prolonged estrogen exposure and its influence on body fat accumulation [14,15,16,17]. Others suggest that late menarche may reflect adverse early-life conditions, such as chronic malnutrition or infectious diseases, which in turn compromise metabolic and cardiovascular health [18,19,20]. It has also been proposed that both early and late menarche are predictors of cardiometabolic disorders [21,22], whereas other reports have found no significant associations [21,22,23]. Moreover, given that early menarche increases the likelihood of excess weight in adulthood, and obesity is a well-established risk factor for hypertension, it is plausible that obesity plays a mediating role in this relationship [24,25,26]. However, evidence supporting this mechanism remains limited.

Importantly, findings in postmenopausal women have been particularly inconsistent, with some investigations reporting no association between age at menarche and hypertension [15,21,22], whereas others suggest that early menarche continues to confer increased cardiometabolic risk in this group [14,17]. This gap is critical, as menopause is characterized by profound hormonal changes that promote central fat redistribution, dyslipidemia, and accelerated cardiovascular risk [27]. Despite the high burden of obesity and hypertension among Mexican women, evidence addressing the role of early reproductive factors such as menarche in this population is scarce, particularly in postmenopausal women. Studying this population could provide valuable insight into how early reproductive factors shape cardiometabolic health in later life, support a life-course approach to cardiovascular research, and contribute to the early identification of women at greater risk. Therefore, we aimed to evaluate the association of early menarche, obesity, and hypertension in postmenopausal women, and to assess whether obesity mediates this relationship.

## 2. Materials and Methods

### 2.1. Design and Study Population

We conducted an analytic cross-sectional study based on a retrospective review of paper-based medical records from women who attended the Climacteric and Menopause Clinic at a tertiary hospital affiliated with the Mexico City Ministry of Health between January 2023 and August 2024. The hospital provides specialized care to individuals without social security coverage.

The analytic sample included 462 postmenopausal women with a clinical diagnosis of menopause, defined as ≥12 consecutive months of amenorrhea in the absence of prior hysterectomy or oophorectomy. Women with documented oncologic disease, thyroid disorders, diabetes mellitus, or chronic kidney disease were excluded. A flow diagram summarizing the selection process of the study population, including the number of excluded records and reasons for exclusion, is presented in the Supplementary Material (Figure S1).

Because this study used a retrospective review of clinical records, all eligible women during the study period were included (census sample). Although no a priori sample size calculation was performed, we estimated the study’s post hoc statistical power for the primary outcome (hypertension). Under a sensitivity assumption using an external baseline prevalence of hypertension of 44% in postmenopausal women [28] and the observed group sizes (early menarche = 214; reference = 248), the study had approximately 80% power to detect a prevalence ratio of 1.30 at α = 0.05.

### 2.2. Study Variables

The dependent variables were obesity and hypertension. Obesity was defined using the body mass index (BMI) recorded in clinical records and classified according to World Health Organization criteria (normal weight: 18.5–24.9 kg/m^2^; overweight: 25.0–29.9 kg/m^2^; and obesity: ≥30.0 kg/m^2^) [29]. Hypertension was defined as a documented medical diagnosis in the clinical record.

The primary independent variable was age at menarche, categorized as early menarche (<12 years) and typical menarche (≥12 years). This cutoff was selected based on previous large-scale epidemiological studies that consistently defined early menarche as occurring before age 12, a threshold associated with increased cardiometabolic and reproductive risk [14,30]. National data also indicate that the mean age at menarche among Mexican women ranges from 11.3 to 12.0 years [31,32], supporting the appropriateness of this threshold for our population.

Covariates included age (in years), marital status (with or without partner), education level (no formal education, primary, lower secondary, upper secondary, or tertiary education), monthly household income (in U.S. dollars), parity (nulliparous, 1–2, or ≥3 live births), alcohol consumption (yes/no), cigarette smoking (yes/no), family history of obesity (yes/no), family history of hypertension (yes/no), use of hormone replacement therapy (ever/never), and age at menopause, classified as early (≤45 years) or typical/late (>45 years).

### 2.3. Data Collection Procedures

Data were obtained retrospectively through a systematic review of paper-based clinical records, as the hospital lacks an electronic health record system. The Clinical Records Department granted authorization to access the files of women with a menopause diagnosis within the specified study period.

Information was extracted using a standardized abstraction form developed for this study. Two trained researchers independently reviewed the records, and any discrepancies were resolved through consensus with a third investigator. To ensure reliability, an independent re-evaluation of 15% of the reviewed records (n = 70) was conducted before applying exclusion criteria. Interobserver agreement was assessed using Cohen’s kappa coefficient, yielding 0.79, which indicates substantial agreement among reviewers.

Completeness and internal consistency of the database were verified prior to analysis. Sixty-six records (6% of all reviewed charts) lacked information on either age at menarche or hypertension and were excluded from the final analyses. A comparison of demographic characteristics between excluded and included participants showed no significant differences, suggesting that the exclusion was unlikely to introduce systematic bias (Table S1).

### 2.4. Statistical Analysis

Descriptive statistics were used to summarize participant characteristics. Continuous variables were tested for normality using the Shapiro–Wilk test and expressed as mean ± SD or median (IQR), as appropriate. Categorical variables were described as frequencies and percentages. Differences by obesity and hypertension status were examined using Pearson’s chi-square test for categorical variables and Student’s *t*-test or Mann–Whitney U test for continuous variables, depending on distribution.

Associations between early menarche and each outcome (obesity and hypertension) were assessed using Poisson regression models with a log link and robust variance to estimate prevalence ratios (PRs) and 95% intervals (CIs). This approach was chosen because odds ratios tend to overestimate the association when the outcome prevalence exceeds 10% [33].

Effect modification by age at menopause was evaluated by including an interaction term between early menarche and menopause age category (≤45 vs. >45 years). The interaction was tested using the Wald test, and a *p*-value ≤ 0.20 was considered suggestive of potential modification. Stratum-specific PRs were reported when applicable [34].

To examine the mediating role of obesity in the association between early menarche and hypertension, we used causal mediation analysis in Stata’s mediate command (version 19.5) within the counterfactual framework. Poisson regression with a log link and robust variance was specified for the outcome (hypertension), and a binomial-logit model was used for the mediator (obesity). We estimated direct and indirect effects and the proportion of the total effect mediated on the PR scale [35].

Additional mediators (age at menopause, alcohol consumption, smoking, and parity) were explored in secondary analyses under the same framework. Sensitivity analyses included re-categorizing age at menarche into <12, 12–15 (reference category), and >15 years, and calculating E-values to quantify the strength of unmeasured confounding required to explain the observed associations [36].

Confounder selection was guided by directed acyclic graphs (DAGs) constructed a priori to identify minimal sufficient adjustment sets (Figure S2) [37,38]. These sets determined the variables included in the multivariable Poisson models reported in the Section 3. To assess the potential impact of unmeasured confounding, we calculated E-values for the point estimate and the lower bound of the 95% CI of the association between early menarche and hypertension. This sensitivity analysis quantifies the minimum strength of association that an unmeasured confounder would need to have with both the exposure and the outcome, above and beyond the measured covariates, to fully explain the observed association [39].

Statistical significance for logistic regression models and hypothesis tests was determined using a *p*-value threshold of <0.05. All analyses were conducted using Stata statistical software, version 19.5 (StataCorp, College Station, TX, USA).

## 3. Results

We reviewed 1002 records and excluded 540 according to prespecified criteria, leaving 462 women for analysis (Figure S2). Table 1 summarizes baseline characteristics. The median age was 51 years (interquartile range, 11 years). Most women were married or living with a partner, and secondary education was the most common level of education. Current alcohol and tobacco use was reported by 10.6% and 9.7% of participants, respectively. A family history of obesity and hypertension was documented in 19.3% and 12.3% of women, respectively. Regarding reproductive history, 53.7% reported age at menarche ≥12 years, and 64.9% experienced menopause after age 45; most participants had one or two children.

Overall, 37.8% of the women in the study had obesity, and 34.9% had hypertension. Women with obesity had significantly higher proportions of early menarche, menopause at or before 45 years, and a family history of obesity or hypertension (*p* < 0.05 for all). Similarly, women with hypertension showed significantly higher proportions of early menarche and a family history of obesity or hypertension (*p* < 0.05). No statistically significant differences were observed for age, marital status, education, parity, or tobacco use.

After adjusting for confounders, women with early menarche had a higher risk of obesity than those with menarche at ≥12 years (adjusted PR = 1.36; 95% CI = 1.08–1.70; *p*-value = 0.009). The association reached statistical significance among women whose menopause occurred at or before 45 years (adjusted PR = 1.54, 95% CI = 1.09–2.16, *p*-value = 0.013), while the estimate among those with menopause after 45 years was in the same direction but not statistically significant (adjusted PR = 1.26, 95% CI = 0.92–1.72, *p*-value = 0.147). The *p*-value for interaction was 0.008, suggesting possible effect modification by age at menopause, although limited power in the stratified analysis may have reduced the precision of estimates (Table 2).

Moreover, early menarche was associated with a significantly higher prevalence of hypertension (adjusted PR = 1.34; 95% CI = 1.06–1.71; *p*-value = 0.015). The E-value for this estimate was 2.02 for the point estimate and 1.31 for the lower 95% CI bound, indicating that only a moderately strong unmeasured confounder could explain away the observed association. Although not statistically significant, the magnitude of the association was higher among women with menopause at or before 45 years (PR = 1.48; 95% CI = 1.02–2.13) compared with those with later menopause (PR = 1.28; 95% CI = 0.93–1.75; *p* for interaction = 0.118; Table 3).

In a sensitivity analysis with three menarche categories (<12, 12–15 [reference category], >15 years; Table 4), early menarche was associated with a higher prevalence of obesity (adjusted PR = 1.38, 95% CI = 1.08, 1.77, *p* = 0.009) and hypertension (adjusted PR = 1.46, 95% CI = 1.13, 1.89, *p* = 0.004) relative to 12–15 years. Conversely, late menarche (>15 years) was associated with a lower risk of obesity (adjusted PR = 0.75, 95% CI = 0.44–1.27, *p* = 0.292) and hypertension (adjusted PR = 0.95, 95% CI = 0.56–1.61, *p* = 0.861). However, these associations were not statistically significant.

**Figure 1:**
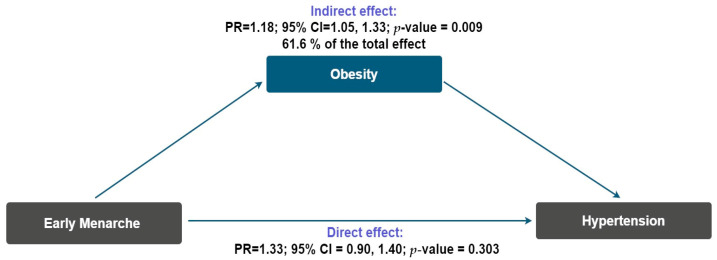
Counterfactual mediation model evaluating the mediating role of obesity in the relationship between early menarche and hypertension in postmenopausal women. PR, Prevalence ratio; CI, confidence interval.

Figure 1 summarizes the mediation analysis. The association between early menarche and hypertension was partially mediated by obesity, which accounted for 61.6% of the total effect. The indirect pathway was statistically significant (PR = 1.18, 95% CI = 1.05–1.33), whereas the direct effect was attenuated and not statistically significant (PR = 1.13, 95% CI = 0.90–1.40). Additionally, we evaluated mediation through age at menopause, alcohol consumption, smoking, and parity. For each mediator, the indirect effect was essentially null (PR ≈ 1.00, not statistically significant), and the proportion mediated ranged from 0.03% for parity to 9.07% for smoking (Table S2).

## 4. Discussion

In this cohort of Mexican postmenopausal women, early menarche (<12 years) was significantly associated with a higher prevalence of both obesity and hypertension. Moreover, obesity appeared to account for a meaningful proportion of this association, suggesting a possible intermediary role in the pathway linking early sexual maturation to later cardiovascular risk. These findings are consistent with evidence from European, Asian, and Latin American cohorts showing that early menarche is associated with increased cardiometabolic risk, although the strength of this association may vary across ethnic groups due to genetic, nutritional, and sociocultural differences [14,40,41,42].

Importantly, our results contribute population-specific data from a Latin American context, where ethnospecific features (such as early-life undernutrition, rapid nutritional transition, and higher prevalence of metabolic disorders) may amplify the long-term cardiometabolic impact of early pubertal timing. This underscores the need to consider regional and ethnic differences when interpreting reproductive and metabolic health associations and highlights the relevance of studying these patterns in underrepresented populations like Mexican women.

Within this context, we observed that 46.3% of participants experienced menarche before the age of 12. This finding is consistent with national reports documenting a progressive decline in the age at menarche, a trend that has become more pronounced in recent decades [31]. Studies conducted in several regions of Mexico have reported mean ages at menarche ranging from 11.3 to 12.0 years, further supporting the consistency of our results [31,32]. Mexico has faced in the past decades one of the highest prevalences of childhood and adolescent overweight and obesity. Excess body fat increases leptin and insulin production, hormones that signal earlier puberty.

In our sample, 37.8% of women had obesity, and 34.9% had hypertension. These prevalences are comparable to national estimates for women, which report obesity in around 41% [43] and hypertension in about 40.2% [4]. Although slightly lower, our estimates still highlight the substantial cardiometabolic burden faced by Mexican women.

We found that women who experienced menarche before the age of 12 were more likely to have hypertension among postmenopausal women. These findings contrast with previous research. For example, a study conducted among Iranian women aged ≥45 years found no significant association between age at menarche and hypertension. In that analysis, age at menarche was treated as a continuous variable in logistic models [21]. Similarly, in South Korean women aged 45–46 years, early menarche was initially associated with a higher likelihood of hypertension; however, the association was attenuated and no longer statistically significant after adjustment for confounders [22]. In addition, a study conducted in Mexico among reproductive-age and postmenopausal women reported a null association (RR = 1.0) when comparing early menarche with later onset [44]. Another study in Brazil reported that in women aged ≥18 years, early menarche was associated with higher odds of hypertension only among those of reproductive age, with no significant association in postmenopausal women [15]. Discrepancies across populations may be partly explained by contextual factors, such as nutrition, genetics, and the stage of epidemiological transition, as well as methodological differences between studies, including the definition of early menarche, which was generally set at <12 years. In addition, ethnospecific features may modulate these associations across populations. Differences in genetic background, early-life nutrition, and sociocultural contexts influence both the timing of menarche and its long-term cardiometabolic consequences. In this sense, our results provide novel population-based evidence from Latin America, a region underrepresented in the literature, emphasizing the importance of examining these associations in diverse ethnic and environmental settings.

The Iranian study modeled age at menarche as a continuous variable, which may have obscured associations at the extremes [21]; the Korean study adjusted for obesity and smoking, variables that can act as mediators rather than confounders, thus attenuating the total effect [22]; and in the Brazilian cohort, the absence of association among postmenopausal women may reflect the stronger impact of age and the coexistence of comorbidities such as diabetes and obesity, which could overshadow the contribution of early menarche to hypertension risk [15].

In contrast, our results are consistent with other investigations. In England, one study reported that early menarche was associated with higher systolic and diastolic blood pressure levels, as well as with an increased likelihood of hypertension among women aged 40 to 80 years [14]. Similarly, a study of Chinese women over 30 years found that those who experienced menarche before 14 years had a higher likelihood of hypertension compared with those whose menarche occurred later, with stratified analyses confirming that the association remained significant and in the same direction among women aged 45 to 65 years [17].

Although the biological mechanisms linking age at menarche to hypertension risk are not fully elucidated, it has been proposed that early menarche prolongs cumulative estrogen exposure and favors higher circulating levels of these hormones throughout reproductive life. Sustained estrogen exposure may reduce sex hormone–binding globulin concentrations, thereby increasing the free and biologically active fractions of these hormones [45]. This imbalance may contribute to endothelial dysfunction, increased sodium retention, and impaired blood pressure regulation. Indeed, blood pressure elevations have been documented in states of high estrogen levels, such as the third trimester of pregnancy, the use of combined oral contraceptives, and estrogen-based hormone replacement therapy [46]. Taken together, these observations support the hypothesis that prolonged hormonal exposure associated with early menarche may represent a plausible mechanism underlying the increased risk of hypertension observed later in life.

In our sensitivity analysis, late menarche (>15 years) appeared to be associated with a lower prevalence of obesity and hypertension. However, these associations were not statistically significant, and the imprecise estimates likely reflect the small sample size in this group (n = 43). Therefore, our results do not provide evidence of a protective effect of late menarche, and the apparent trend should be interpreted with caution. Previous population-based studies have suggested a protective effect of late menarche against hypertension. For example, a Brazilian study found an inverse association between age at menarche and both systolic and diastolic blood pressure levels [47], while a study in Chinese women over 50 years old reported that those with menarche before 12.5 years had a higher probability of hypertension compared to those whose menarche occurred at ≥14.5 years [24]. Moreover, several investigations have reported that both very early and very late menarche are linked to increased cardiovascular risk [30,48]. This suggests that extremes of age at menarche may reflect underlying biological disruptions with long-term implications for cardiometabolic health.

In this study, we also found that early menarche was significantly associated with obesity in postmenopausal women. This observation is consistent with previous research showing that earlier pubertal timing is associated with a higher risk of excess adiposity throughout the life course [49]. Longitudinal studies have demonstrated that girls who experience menarche at younger ages are more likely to accumulate greater body mass index and waist circumference during adulthood [49,50], reinforcing the role of early sexual maturation as a determinant of long-term obesity risk.

Our findings suggest that obesity may play a potential intermediary role in the relationship between age at menarche and hypertension. Previous studies have proposed that adiposity indicators, such as body mass index and waist circumference, partially explain this association [24,25,26]. In line with this evidence, the present study provides results consistent with a possible pathway involving obesity in a Mexican population, where the burden of obesity and cardiometabolic disease is particularly high. Although the indirect effect observed supports a biologically plausible mechanism, this interpretation should be approached with caution, given the potential for unmeasured confounding between obesity and hypertension.

Early menarche entails prolonged exposure to estrogens, which promotes fat deposition in peripheral adipose tissue [51,52,53]; in turn, obesity may contribute to elevated blood pressure through multiple mechanisms, including insulin resistance, increased sympathetic activity, aldosterone secretion, sodium and water retention, higher cardiac output, and endothelial dysfunction mediated by adipokines such as leptin and adiponectin [54]. Beyond these pathways, excess adiposity also fosters a pro-inflammatory milieu that exacerbates vascular injury and raises blood pressure.

Taken together, these findings support a biologically coherent framework linking early reproductive events and cardiometabolic outcomes, while underscoring the potential value of strategies targeting obesity prevention among women with early menarche to mitigate future cardiovascular risk.

Finally, when examining the potential modifying effect of age at menopause, the associations between early menarche and both obesity and hypertension tended to be stronger among women with early menopause, suggesting that reproductive life expectancy may play a role in shaping cardiovascular trajectories. A shorter reproductive life expectancy may reduce cumulative estrogen exposure, accelerating vascular aging, endothelial dysfunction, and metabolic disturbances that plausibly increase the likelihood of obesity and hypertension. However, these analyses should be interpreted with caution, as the exact age at diagnosis was not consistently available for both conditions, which could have introduced some degree of misclassification.

From a practical perspective, these findings underscore the importance of incorporating reproductive history, particularly age at menarche, into cardiovascular risk assessment in women. Identifying those with early menarche could allow clinicians to recognize individuals at greater risk and to implement preventive measures, such as lifestyle counseling or early blood pressure monitoring, throughout adulthood and postmenopause. Such an approach could strengthen life-course strategies to reduce the burden of cardiometabolic disease among women.

### Limitations and Strengths

Our results have limitations that should be considered when interpreting them. The cross-sectional design does not allow for establishing causality; therefore, the associations reported should be interpreted with caution. However, because menarche typically precedes the development of obesity and hypertension, the identified relationships are temporally coherent and biologically plausible. In addition, participants were not selected based on their obesity or hypertension status, which reduces the likelihood of selection bias. A small proportion of records (6%) were excluded because they lacked data on either age at menarche or sufficient information to confirm or rule out hypertension. These excluded cases did not differ demographically from included participants; therefore, our findings are unlikely to be affected by selection bias. Reliance on medical records may also entail some degree of measurement error; however, data were collected by two trained researchers using a standardized abstraction form, which minimized the likelihood of inaccuracies. Moreover, we have no reason to assume that any remaining errors were systematically related to the study outcomes, and potential misclassification is therefore expected to be non-differential.

Moreover, although we adjusted for several relevant sociodemographic and clinical covariates, residual confounding cannot be ruled out entirely. For instance, we lacked information on early-life factors such as body mass index during puberty or socio-economic conditions in childhood and adolescence, both of which are known to influence age at menarche, through their effects on growth, energy balance, and pubertal timing, and to shape lifelong cardiometabolic risk by affecting adiposity, dietary patterns, and metabolic function. To assess this limitation, we quantified the potential impact of residual confounding using E-values. For the association between early menarche and hypertension (PR = 1.34, 95% CI 1.06–1.71), the E-value was 2.02 for the point estimate and 1.31 for the lower 95% CI bound. This suggests that an unmeasured confounder would need to be associated with both early menarche and hypertension, with a risk ratio of at least 1.31 beyond the measured covariates, to explain the observed association fully. Thus, our results appear reasonably robust to moderate unmeasured confounding.

Nevertheless, the lack of detailed information on early-life nutritional status and life-course socioeconomic trajectories may have introduced residual confounding beyond what the E-value analysis can capture. These factors could influence both the timing of menarche and subsequent cardiometabolic risk through intergenerational and developmental mechanisms. Therefore, our findings should be interpreted with caution, and future longitudinal studies incorporating early-life measures are warranted to clarify these pathways and strengthen causal inference.

Furthermore, our mediation analysis assumes that there are no unmeasured confounders in the mediator-outcome relationship. In our setting, factors such as diet quality, physical inactivity/sedentary behavior, sleep disturbances, and chronic psychosocial stress could affect both obesity and blood pressure, but were not fully captured. If present, these factors would likely bias the indirect effect toward the null hypothesis (i.e., overestimating mediation). Hence, mediation findings should be interpreted with caution and considered hypothesis-generating. Another limitation is that the late-menarche stratum (>15 years) included a relatively small number of participants, which limited the statistical power and precision of the sensitivity analysis; estimates for this group should therefore be interpreted with caution. In addition, the study population was derived from a single tertiary care center belonging to the Mexican Ministry of Health, which primarily serves individuals without social security coverage. This feature implies that participants in our study may differ from the general Mexican population, as individuals with social security in Mexico tend to have higher educational attainment and household income. Therefore, some degree of socioeconomic selection cannot be ruled out, and the findings should be generalized with caution. Nonetheless, the biological mechanisms linking early menarche, obesity, and hypertension are not expected to differ substantially across populations, which supports the plausibility of our findings beyond the study setting. Future studies with longitudinal designs and larger, population-based samples are warranted to characterize these associations better and confirm potential nonlinear patterns.

## 5. Conclusions

The results of this study show that early menarche is independently associated with obesity and hypertension in postmenopausal women. We also observed that age at menopause modified the magnitude of these associations and that obesity partially mediated the relationship between age at menarche and hypertension. These findings add to the evidence highlighting the influence of reproductive events on women’s metabolic health and provide novel evidence in the Mexican population, where studies on this topic have been limited. Considering that obesity and hypertension are highly prevalent among Mexican women, preventive measures should prioritize groups with greater susceptibility, such as those with early menarche. Identifying early menarche as a possible risk marker could be incorporated into the clinical evaluation of women in the post-reproductive stage. Our findings reinforce the importance of monitoring adiposity and blood pressure from early life and promoting healthy habits throughout the life course, beginning in adolescence and continuing into adulthood and menopause.

## Figures and Tables

**Table 1:** Participant characteristics stratified by obesity and hypertension status.

Features	Total (n = 462)	Obesity			Hypertension		
		No (n = 287)	Yes (n = 175)	*p*-Value ^a^	No (n = 296)	Yes (n = 166)	*p*-Value ^a^
Age (in years)							
Median (IQR)	51 (11)	52 (10)	52 (12)	0.854	51.5 (10)	53 (15)	0.085
Marital status, n (%)							
No partner	98 (21.2)	56 (57.1)	42 (42.9)	0.252	55 (56.1)	43 (43.9)	0.065
With partner	364 (78.8)	231 (63.5)	133 (36.5)		241 (66.2)	123 (33.8)	
Education level, n (%)							
No formal education	37 (8.0)	19 (51.4)	18 (48.6)	0.095	20 (54.0)	17 (46.0)	0.172
Primary education	62 (13.4)	35 (56.5)	27 (43.5)		33 (53.2)	29 (46.8)	
Lower secondary education	153 (33.1)	91 (59.5)	62 (40.5)		100 (65.4)	53 (34.6)	
Upper secondary education	186 (40.3)	129 (69.4)	57 (30.6)		127 (68.3)	59 (31.7)	
Tertiary education	24 (5.2)	13 (54.2)	11 (45.8)		16 (66.7)	8 (33.3)	
Monthly household income ^b^							
Median (IQR)	526.4 (163.1)	528.3 (159.3)	529.5 (181.4)	0.629	535.1 (167.3)	524.1 (168.6)	0.085
Parity, n (%)							
Nulliparous	16 (3.5)	12 (75.0)	4 (25.0)	0.397	11 (68.7)	5 (31.3)	0.864
1–2	293 (63.4)	185 (63.1)	108 (36.9)		189 (64.5)	104 (35.5)	
≥3	153 (33.1)	90 (58.8)	63 (41.2)		96 (62.8)	57 (37.2)	
Alcohol consumption, n (%)							
No	413 (89.4)	263 (63.7)	150 (36.3)	0.045	269 (65.1)	144 (34.9)	0.988
Yes	49 (10.6)	24 (49)	25 (51)		27 (55.1)	22 (44.9)	
Cigarette smoking, n (%)							
No	417 (90.3)	259 (62.1)	158 (37.9)	0.141	275 (65.9)	142 (34.1)	0.731
Yes	45 (9.7)	28 (62.2)	17 (37.8)		21 (46.7)	24 (53.3)	
Family history of obesity, *n* (%)							
No	373 (80.7)	247 (66.2)	126 (33.8)	0.001	267 (71.6)	106 (28.4)	0.001
Yes	89 (19.3)	40 (44.9)	49 (55.1)		29 (32.6)	60 (67.4)	
Family history of hypertension, *n* (%)							
No	405 (87.7)	269 (66.4)	136 (33.6)	0.001	269 (66.4)	136 (33.6)	0.005
Yes	57 (12.3)	18 (31.6)	39 (68.4)		27 (47.4)	30 (52.6)	
Age of menarche, *n* (%)							
≥12 years	248 (53.7)	168 (67.7)	80 (32.3)	0.007	172 (69.3)	76 (30.7)	0.011
<12 years	214 (46.3)	119 (55.6)	95 (44.4)		124 (57.9)	90 (42.1)	
Age of menopause, n (%)							
>45 years	288 (62.3)	194 (67.4)	94 (32.6)	0.003	188 (65.3)	100 (34.7)	0.486
≤45 years	174 (37.7)	93 (53.5)	81 (46.5)		108 (62.1)	66 (37.9)	

Abbreviations: IQR, interquartile range. ^a^ Comparing subjects by obesity and hypertension status using Pearson’s chi-squared test for categorical variables and the Mann–Whitney U test for the difference in medians. ^b^ American dollars.

**Table 2:** Adjusted prevalence ratios for the association between age at menarche and obesity, stratified by age at menopause.

Age at Menarche	Overall		Age at Menopause				
			≤45 Years (n = 288)		>45 Years (n = 174)		
	PR (CI 95%) ^a^	*p*-Value	PR (CI 95%) ^a^	*p*-Value	PR (CI 95%) ^a^	*p*-Value	*p*-Value for Interaction
≥12 years	Ref.	-	Ref.	-	Ref.		0.008
<12 years	1.36 (1.08–1.70)	0.009	1.54 (1.09–2.16)	0.013	1.26 (0.92–1.72)	0.147	

Abbreviations: Ref, reference; PR, prevalence ratio; CI, confidence interval. ^a^ All models were adjusted for age, education, monthly household income, and family history of obesity.

**Table 3:** Adjusted prevalence ratios for the association between age at menarche and hypertension, stratified by age at menopause.

Age at Menarche	Overall		Age at Menopause				
			≤45 Years (n = 288)		>45 Years (n = 174)		
	PR (CI 95%) ^a^	*p*-Value	PR (CI 95%) ^a^	*p*-Value	PR (CI 95%) ^a^	*p*-Value	*p*-Value for Interaction
≥12 years	Ref.	-	Ref.	-	Ref.		0.118
<12 years	1.34 (1.06–1.71)	0.015	1.48 (1.02–2.13)	0.036	1.28 (0.93–1.75)	0.123	

Abbreviations: Ref, reference; PR, prevalence ratio; CI, confidence interval. ^a^ All models were adjusted for age, education, monthly household income, and family history of hypertension.

**Table 4:** Sensitivity analysis: adjusted prevalence ratios for the association between age at menarche (three categories) and obesity and hypertension.

Age at Menarche	n (%)	Obesity		Hypertension	
		PR (CI 95%) ^a^	*p*-Value	PR (CI 95%) ^a^	*p*-Value
12–15 years	205 (44.40)	Ref.	-	Ref.	-
<12 years	214 (46.30)	1.38 (1.08–1.77)	0.009	1.46 (1.13–1.89)	0.004
<15 years	43 (9.30)	0.75 (0.44–1.27)	0.292	0.95 (0.56–1.61)	0.861

Abbreviations: Ref, reference; PR, prevalence ratio; CI, confidence interval. ^a^ All models were adjusted for age, education, monthly household income, and family history of hypertension.

## Data Availability

The data that support the findings of this study are openly available in Mendeley Data at doi: 10.17632/htd7rzxtj7.1.

## Supplementary Materials

The following supporting information can be downloaded at: https://www.mdpi.com/article/10.3390/epidemiologia6040086/s1. Figure S1: Study selection flowchart; Figure S2: Directed acyclic graph of early menarche, obesity, and hypertension; Table S1: Characteristics of included and excluded records in the study; Table S2: Secondary Mediation Analyses.

## Abbreviations

The following abbreviations are used in this manuscript:

PR	Prevalence Ratio
CI	Confidence Interval
DAG	Directed Acyclic Graph
BMI	Body Mass Index
