# Supplementary material for: Early Menarche and Hypertension Among Postmenopausal Women: The Mediating Role of Obesity
Source: Epidemiologia (Basel). 2025 Dec 2;6(4):86. doi: 10.3390/epidemiologia6040086 (PMC12732153; doi:10.3390/epidemiologia6040086)
Supplement: Supplementary file 1 [file epidemiologia-06-00086-s001.zip › Supplementary Table S1.pdf]

Table S1. Characteristics of included and excluded records in the study

Features	Included (n=462)	Excluded (n=66)	p-value[a]
**Age (in years)**			
Median (IQR)	51 (11)	52 (11)	0.854
**Marital status, n (%)**			
No partner	98 (21.2)	15 (22.7)	0.911
With partner	364 (78.8)	51 (80.3)	
**Education level, n (%)**			
No formal education	37 (8.0)	5 (7.6)	0.749
Primary education	62 (13.4)	7 (10.6)	
Lower secondary education	153 (33.1)	21 (31.8)	
Upper secondary education	186 (40.3)	27 (40.9)	
Tertiary education	24 (5.2)	6 (9.1)	
**Monthly household income** [b]			
Median (IQR)	526.4 (163.1)	527.5 (180.4)	0.619
**Parity, n (%)**			
Nulliparous	16 (3.5)	5 (25.0)	0.397
1-2	293 (63.4)	31 (36.9)	
≥3	153 (33.1)	30 (41.2)	
**Alcohol consumption, n (%)**			
No	413 (89.4)	57 (86.6)	0.562
Yes	49 (10.6)	9 (13.4)	
**Cigarette smoking, n (%)**			
No	417(90.3)	58 (90.0)	0.999
Yes	45 (9.7)	6 (9.1)	
**Family history of obesity, *n* (%)**			
No	373 (80.7)	49 (78.8)	0.612
Yes	89 (19.3)	14 (21.2)	
**Family history of hypertension, *n* (%)**			
No	405 (87.7)	55 (83.3)	0.327
Yes	57 (12.3)	11 (16.7)	
**Age of menopause, n (%)**			
>45 years	288 (62.3)	66 (61.7)	0.912
≤ 45 years	174 (37.7)	41 (38.3)	

Abbreviations: IQR, interquartile range.

[a] Comparing subjects using Pearson's chi-squared test for categorical variables and the Mann–Whitney U test for the difference in medians.

[b] American dollars
