# Supplementary material for: Early Menarche and Hypertension Among Postmenopausal Women: The Mediating Role of Obesity
Source: Epidemiologia (Basel). 2025 Dec 2;6(4):86. doi: 10.3390/epidemiologia6040086 (PMC12732153; doi:10.3390/epidemiologia6040086)
Supplement: Supplementary file 1 [file epidemiologia-06-00086-s001.zip › Supplementary_Table S2 _Mediation.pdf]

Table S2. Secondary Mediation Analyses

Mediator	Indirect effect: PR (95% CI)[a]	P-value	Proportion mediated (%)[a]
**Age at menopause**	1.00 (0.98, 1.06)	0.431	2.68
**Alcohol consumption**	1.00 (0.99, 1.01)	0.617	1.17
**Smoking**	1.02 (0.98, 1.06)	0168	9.07
**Parity**	1.00 (0.99, 1.00)	0.978	0.03

Abbreviations: PR, prevalence ratio; CI, confidence interval.

[a] Analyses were conducted under the counterfactual causal mediation framework.
