# Supplementary figures and images for: Early Menarche and Hypertension Among Postmenopausal Women: The Mediating Role of Obesity
Source: Epidemiologia (Basel). 2025 Dec 2;6(4):86. doi: 10.3390/epidemiologia6040086 (PMC12732153; doi:10.3390/epidemiologia6040086)

**Figure S1.** Study selection flowchart.

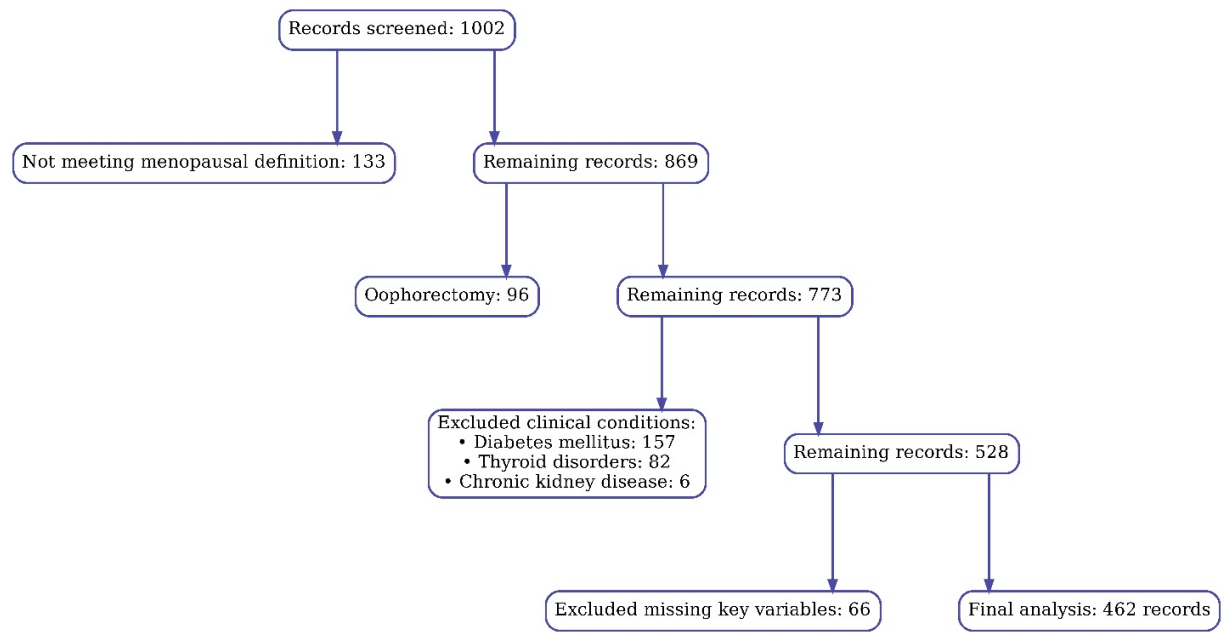

Supplement: Supplementary file 1 [file epidemiologia-06-00086-s001.zip › Supplementary Figure 1.pdf]

Figure S2. Directed acyclic graph of early menarche, obesity, and hypertension

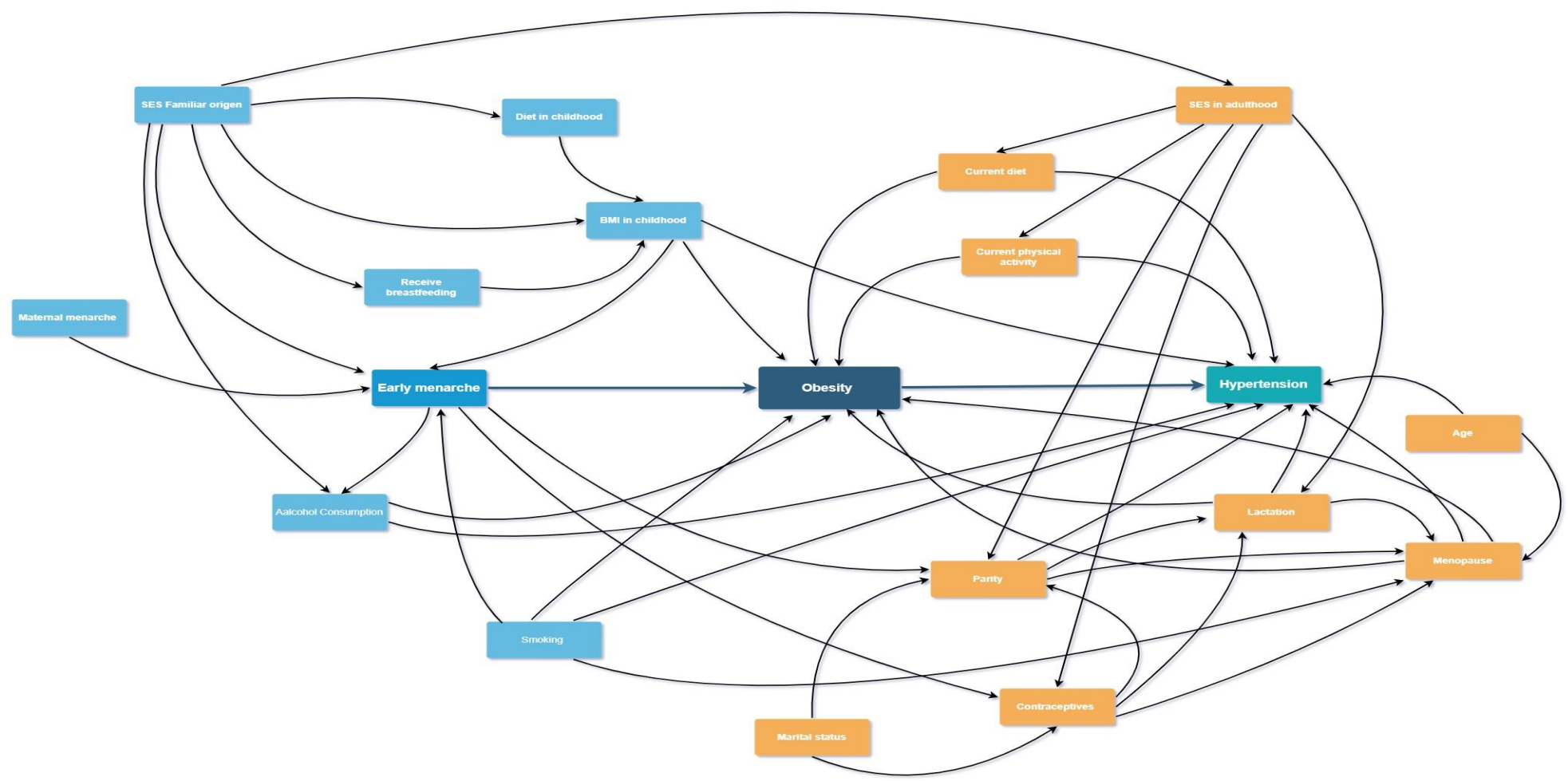

Supplement: Supplementary file 1 [file epidemiologia-06-00086-s001.zip › Supplementary Figure 2.pdf]
